# Protocol for synthesizing, implanting, and using polydimethylsiloxane as skull replacement in mice for imaging, electrophysiology, and optogenetics

**DOI:** 10.1016/j.xpro.2025.103964

**Published:** 2025-07-22

**Authors:** Kengo Takahashi, Gerjan Huis in ‘t Veld, Davide Benedetti, Jun-Ying Wang, Samuel Pontes Quero, Rafael Yuste, Cyriel M.A. Pennartz, Umberto Olcese

**Affiliations:** 1 Swammerdam Institute for Life Sciences, University of Amsterdam, 1098XH Amsterdam, the Netherlands; 2 Amsterdam Brain and Cognition, University of Amsterdam, 1098XH Amsterdam, the Netherlands; 3 Department of Translational Research and of New Surgical and Medical Technologies, University of Pisa, 56124 Pisa, Italy; 4 NeuroTechnology Center, Department of Biological Sciences, Columbia University, New York City, NY 10027, USA

**Keywords:** Microscopy, Neuroscience, Cognitive Neuroscience, Behavior

## Abstract

Many techniques to record and manipulate neuronal activity across large portions of the vertebrate brain are now available. However, few effective approaches enable both optical and mechanical access to the brain. Here, we present a protocol for synthesizing, implanting, and using polydimethylsiloxane (PDMS) windows as skull replacements for chronic wide-field and two-photon calcium imaging in mice. We also describe steps for performing viral injections and multi-site silicon probe implantation.

## Highlights

• Guidance on synthesis of PDMS windows for skull replacement
• Instructions for implantation of PDMS windows as skull replacement
• Steps for optogenetic viral injection and Neuropixels probe insertion
• Assessment of cortical activity via wide-field and two-photon calcium imaging

## Abstract (editor highlights)

Publisher’s note: Undertaking any experimental protocol requires adherence to local institutional guidelines for laboratory safety and ethics.

## Graphical abstract

**Figure:**
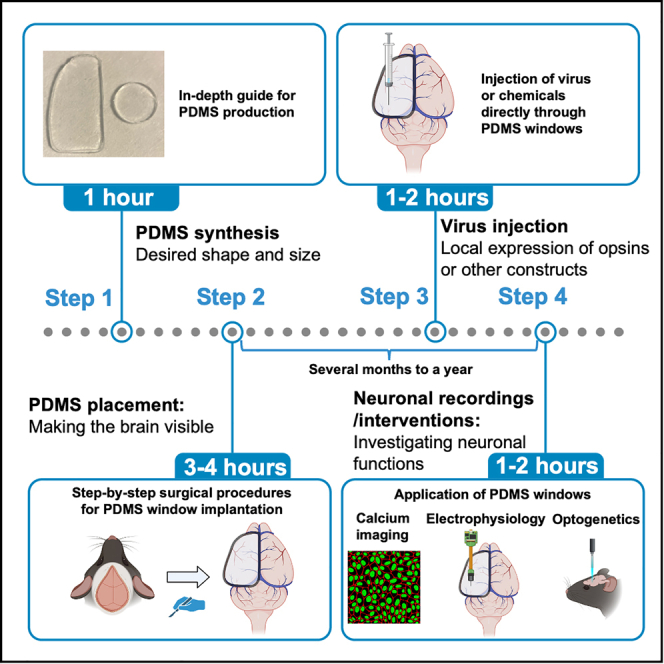

## Before you begin

Recent advancements in neural activity recording techniques, such as calcium imaging and electrophysiology, as well as in neural control methods like optogenetics, have significantly improved our ability to examine the interactions of neuronal dynamics across multiple brain regions during perceptual tasks.^1^^,^^2^^,^^3^^,^^4^ These multimodal neuronal imaging approaches are particularly relevant in studies aimed at identifying and manipulating specific neuronal populations involved in sensory-motor and cognitive transformations, a process that involves interactions between multiple areas across the whole cortex such as conscious visual processing.^5^^,^^6^^,^^7^ To perform neural imaging across the whole cortex, the skull needs to be made transparent. Glass windows have long been used to enable functional, neuron-level imaging,^8^ but their rigidity makes them unsuitable for replacing the whole dorsal skull in long-term use over several months. Skull-clearing techniques^9^ do not suffer from this limitation, but, similarly to glass windows, do not allow mechanical access to the brain (e.g., for viral injections) without opening a craniotomy, a procedure which reduces transparency and increases the risk of infections. Finally, 3D-printed skull replacements have emerged to enable multi-site silicon probe recordings,^1^^,^^10^ but are not currently suitable for large-scale imaging. Flexible polymeric windows,^11^^,^^12^^,^^13^ e.g. made of polydimethylsiloxane (PDMS), potentially allow to overcome the limitations of the previously mentioned approaches, but standardized protocols to employ them are currently not available. To address this, we provide comprehensive, step-by-step instructions for synthesizing biologically compatible PDMS for use in neuronal imaging. We detail the surgical procedure for PDMS implantation, followed by methods for viral or chemical injections through the PDMS window. Finally, the protocol demonstrates the long-term stability for several months to a year and quality of the implanted PDMS windows via examples of how wide-field and two-photon neural imaging can be achieved and integrated with multiple techniques, including electrophysiology and optogenetics. This protocol is primarily designed for mice. However, the presented technique is adaptable to neuronal imaging of other species, such as rats,^11^ ferrets,^14^ and birds,^15^ by adjusting the size and shape of the PDMS window to suit specific experimental requirements. Thus, this protocol offers a comprehensive potential for investigating brain functions in a variety of neuroscience methodologies.

### Institutional permissions

All animal experiments described here complied to international and national legislation and were approved by the Dutch Commission for Animal Experiments and by the Animal Welfare Body of the University of Amsterdam.

### Additional resources

This study was pre-registered at bioRxiv. doi: https://doi.org/10.1101/2025.04.15.648966.

## Key resources table

**Table:** 

REAGENT or RESOURCE	SOURCE	IDENTIFIER
**Bacterial and virus strains**		

AAV8-hSyn1-Jaws_KGC_tdTomato_ER2-WPRE-bGHp(A)	Viral Vector Facility - Universität Zürich	v988-8

**Chemicals, peptides, and recombinant proteins**		

Baytril	Bayer Animal Health GmbH	E110564
Carprofen	Dechra	REG NL 112210
Eye ointment (Lubrithal)	Dechra	321/00/12PUVPT
Lidocaine 2%	Dechra	REG NL 8632
Saline (0.9% sodium chloride)	Fresenius Kabi	1310221
Simplex Rapid	Kemdent	ACR920
Betadine	Mylan	RVG 01331
Dexamethasone	Schnelldorf Distribution	PHR1768-1G
Alexa Fluor 488 hydrazide	Thermo Fisher Scientific	A10436
Dental cement	Sun Medical Co., Ltd.	K058E
Isoflurane	Zoetis	B506

**Experimental models: Organisms/strains**		

Mouse: Rasgrf2-dCre (JAX #022864) x Ai148D (JAX #030328)	The Jackson Laboratory	JAX #022864 (Rasgrf2-dCre); JAX #030328 (Ai148D)

**Recombinant DNA**		

AAV-Syn-Jaws-KGC-tdTomato-ER2	Addgene (Chuong et al.^16^)	Plasmid #153535

**Software and algorithms**		

MATLAB-based acquisition software and a pre-processing pipeline for wide-field imaging	Couto et al.^2^	https://github.com/churchlandlab/WidefieldImager
Open Ephys GUI for Neuropixels Probes	Open Ephys	https://github.com/open-ephys/plugin-GUI
Kilosort3	Pachitariu et al.^17^	https://github.com/MouseLand/Kilosort/tree/v3.0
suite2p	Pachitariu et al.^18^	https://github.com/MouseLand/suite2p
ThorImageLS 4.3	Thorlabs	https://www.thorlabs.com/newgrouppage9.cfm?objectgroup_id=9072#ad-image-0

**Other**		

0.3-mL syringes (BD Micro-Fine + Demi)	BD	324826
25 gauge needle (BD Microlance 3)	BD	300600
30 gauge needle (BD Microlance 3)	BD	304000
Vacuum pump	BILTEC (Polyestershoppen BV)	BT115VM
Blue bandpass filter	Chroma	ET470/40x
Gauze	Cutisoft	45846-00
SYLGARD 184 Silicone Elastomer Kit	Dow, Inc.	1060040_S1
Nanoject II microinjectors	Drummond Scientific	# 490007-164
Nanoject glass capillaries	Drummond Scientific	3-000-203-G/X
Flexdym 250 μm	Eden Tech	M-FS
Green bandpass filter	Edmund Optics	86-963
Green dichroic filter (50 mm Square 45°)	Edmund Optics	47-950
Dental drill	Eickemeyer	
Isoflurane vaporizer	Eickemeyer	213261
Petri dish (90 × 16 mm)	Eurofysica	016610
Absorbable hemostatic gelatin sponge (Spongostan Dental)	Ferrosan Medical Devices	MS0005
Fine scissors	Fine Science Tools	14094-11
Forceps	Fine Science Tools	11251-35
Petri dish (145 × 20 mm)	Greiner Bio-One	639102
0.5 mm diameter drill tip	Hager & Meisinger GmbH	310204001001005
0.9 mm diameter drill tip	Hager & Meisinger GmbH	310204001001009
Cotton swab	Heinz Herenz	TM1301105
Super glue (Pattex ULTRA GEL super glue)	Henkel	5410091669058
Neuropixels 1.0 probe	IMEC (Jun et al.^19^)	PRB_1_4_0480_1_C
Neuropixels 2.0 probe	IMEC (Steinmetz et al.^20^)	NP2014
Biopsy punch	Kai Medical	BP-15F
Vacuum Dome (8 L)	Kartell (Polyestershoppen BV)	N/A
Stereotaxic device (Harvard Apparatus U-frame Stereotaxic Instrument)	Multi Channel Systems	751810
Nikon AF-D 105MM F/2.0 DC Occasion	Nikon	018208019328
5-mL syringes	Nipro	SY3-5LC-GEC
1-mL syringes	Servoprax GmbH	I3040200
Sterile absorbent swabs (Sugi sponge points)	Questalpha GmbH & Co. KG	31602
Ultra gel (thick glue)	Pattex	5410091669058
Twinsil (two-component silicone glue)	Picodent	13001000
85 mm F1.4 Aspherical fixed lens	Rokinon	85M-C
pco.edge 5.5 USB sCMOS camera	PCO AG (SDVision)	pco.edge 5.5 M-AIR-CH-BLU-P
Femtosecond laser (920 nm)	Spark Lasers	ALCOR 920
Scalpel blades	Swann-Morton	0205
Collimator with Ø2″ Lens, AR coating: 350–700 nm	Thorlabs	SM2F32-A
Mounted LED (470 nm)	Thorlabs	M470L5
Multiphoton Imaging microscope	Thorlabs	Bergamo II
Nikon Plan Fluorite Water Dipping Objective (16×)	Thorlabs	N16XLWD-PF
T-Cube LED Driver, 1200 mA Max Drive Current	Thorlabs	LEDD1B
Head bar	Zenodo	https://doi.org/10.5281/zenodo.15577657
Head bar holder	Zenodo	https://doi.org/10.5281/zenodo.15577657
Metal ring	Zenodo	https://doi.org/10.5281/zenodo.15577657
Plastic cover for metal ring	Zenodo	https://doi.org/10.5281/zenodo.15577657

## Materials and equipment

This protocol employs a standardized neural recording system, featuring Neuropixels probes for electrophysiological recordings,^19^^,^^20^ a pco.edge 5.5 USB sCMOS camera (PCO AG) for wide-field calcium imaging,^2^ and a multiphoton imaging microscope (Bergamo II, Thorlabs) for two-photon imaging.

## Step-by-step method details

### PDMS synthesis protocol

**Timing: 1 h (2–3 days waiting)**

**Figure 1:**
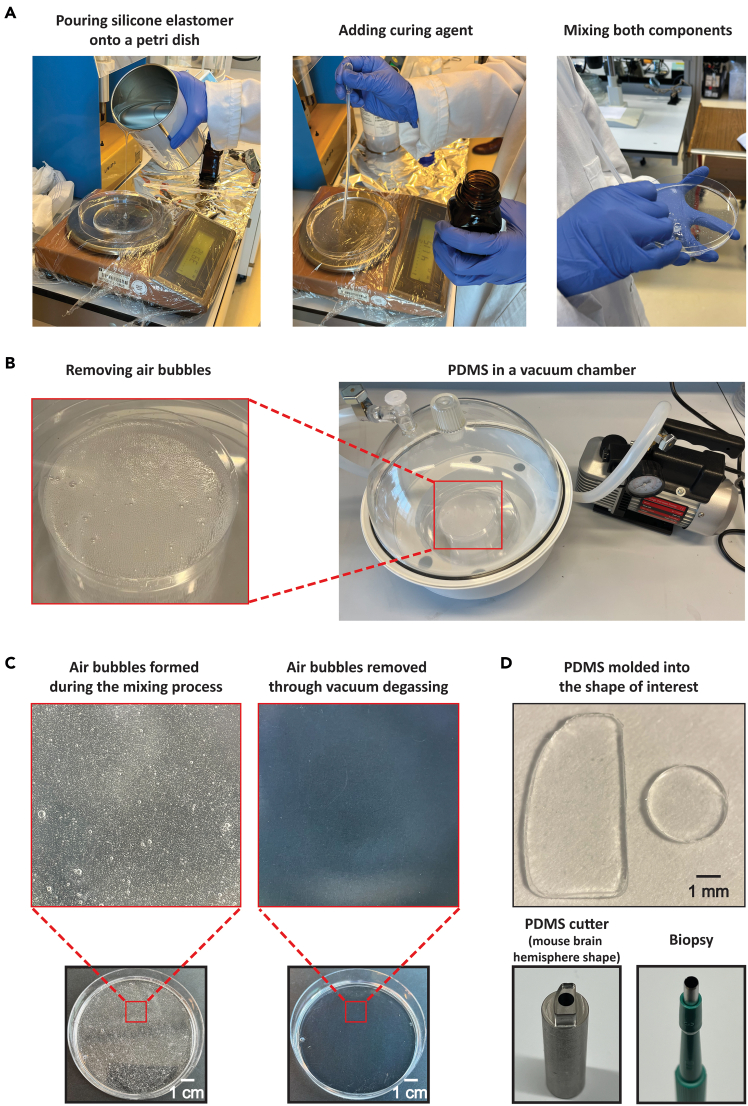
PDMS fabrication process (A) Weighing the silicone elastomer (left) and curing agent (middle), followed by mixing both components (right). (B) Removing air bubbles using a vacuum chamber. (C) Side-by-side comparison of PDMS before (left) and after (right) bubble removal. (D) PDMS molded into specific shapes, such as a hemisphere of the mouse brain using a PDMS cutter (left) and a circle using a biopsy (right).

***Note:*** In this example, a 145 × 20 mm Petri dish was used, but the size is not crucial at this stage, as the primary goal is to mix the two components. A larger dish can be used for larger batches, while a smaller one is suitable for smaller portions. To prevent spills and simplify cleanup, it is advisable to place a layer of plastic wrap on the scale before pouring the silicone elastomer.

1. Add 40 g of Sylgard 184 polymeric base (Dow Inc.) into a plastic Petri dish (Figure 1A, left).

2. Add 4 g of Sylgard 184 curing agent (Dow Inc.), maintaining a 10:1 ratio between the base and curing agents (Figure 1A, middle).
3. Mix vigorously using a sterile stick for 5–10 min until the components are homogeneously blended (Figure 1A, right). During this process, a significant amount of air bubbles will form in the mixture (Figure 1C, left).
4. Apply a vacuum (pressure: 0.05 mbar at room temperature) for 5–10 min, then slowly open a valve to reintroduce air into the chamber (Figure 1B, right). The bubbles in the liquid will rise to the surface (Figure 1B, left) and then disappear.

**CRITICAL:** Rapid valve opening may disrupt the mixture.

5. Repeat Step 4 multiple times until the majority of air bubbles (∼95%) has been removed.
6. Transfer the mixture to another plastic Petri dish to achieve the desired thickness, which was targeted between 300–400 μm.

***Note:*** This range was chosen to ensure that the PDMS was not too thin, which could lead to structural damage and potential harm to the brain, while also maintaining sufficient thinness for effective imaging and ease of making an opening in the PDMS window for electrophysiology later (see the expected outcomes section, Figures 4A and 5A for details). For instance, **2.89 g** of the mixture was placed in a new Petri dish with a diameter of 8.5 cm, yielding a theoretical PDMS thickness of approximately 500 μm. However, the final PDMS thickness in the area intended for use is typically about 100 μm thinner than the theoretical value, due to the accumulation of PDMS along the edges of the dish, caused by its high viscosity during solidification. Thus, adjustments may be required to achieve a more precise thickness.

**CRITICAL:** The adopted container must be smooth and flat, such as a Petri dish, to ensure the surface of the PDMS also becomes smooth, preventing optical distortions (troubleshooting 1).

7. Repeat Step 4 until all visible air bubbles are eliminated (Figure 1C, right).

***Note:*** If air bubbles still remain, extend the vacuum duration beyond 10 min. Optionally, leave the PDMS in the vacuum for 30 min to 1 h, and repeat the process as necessary until the bubbles are fully eliminated.

8. Leave the Petri dish on a flat surface and allow the PDMS to solidify fully (∼2 days). Alternatively, the process can be accelerated by applying heat using an oven.

**CRITICAL:** It is crucial to select a location with minimal slope, as gradients can result in uneven PDMS thickness across different areas of the petri dish. Once the PDMS has fully solidified, it is important to measure its thickness to determine if it is consistent. If not, use PDMS from regions where the thickness falls within the desired range (ideally between 300–500 μm).

9. On the day of PDMS placement surgery, cut the solidified PDMS into the desired shape – e.g., circular, hemispherical, etc. (Figure 1D). Using a mold, such as a biopsy punch, can assist with shaping, though a surgical knife or scissors can also be used.

***Note:*** In the example illustrated in Figure 1D (bottom left), the mold was fabricated by milling after being designed for 3D printing. As described in this procedure, 40 g of Sylgard 184 polymeric base and 4 g of Sylgard 184 curing agent are used to prepare 11 Petri dishes (Dimensions: 90 x 16 mm, with a measured diameter of 85 mm for the plate used to pour PDMS) of PDMS. From each Petri dish, approximately 50 PDMS windows can be obtained.

### Surgical protocol for PDMS placement

**Timing: ∼3–4 h**

**Figure 2:**
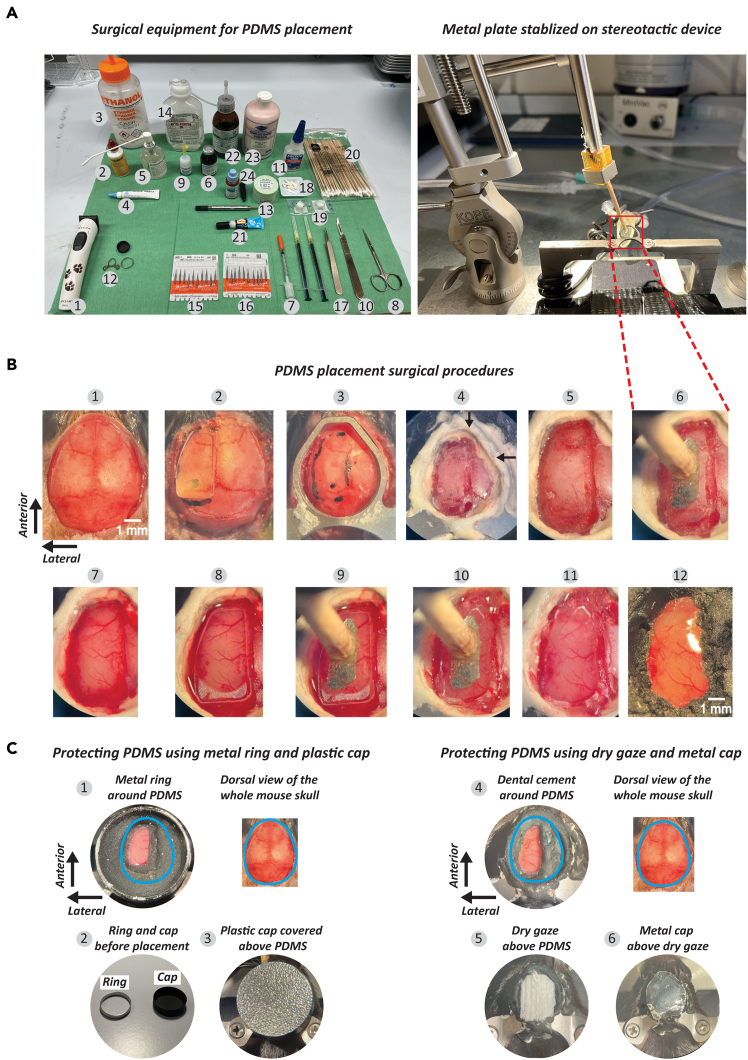
Surgical procedure for PDMS implementation (A) Surgical Setup and Equipment: Displays the surgical instruments (left) and the setup (right) required for PDMS placement. (1) Hair shaver. (2) Povidone-iodine. (3) 70% Ethanol. (4) Eye ointment. (5) Lidocaine. (6) Carprofen. (7) 0.3 ml syringes. (8) Surgical scissors. (9) Vetbond. (10) Surgical scalpel. (11) Super glue. (12) Head bar. (13) Dental cement. (14) Saline. (15) Drill tip (0.9 mm). (16) Drill tip (0.5 mm). (17) Tweezers. (18) Triangular sponges. (19) Gelatin sponge. (20) Cotton swab. (21) Thick glue. (22) Catalyzer for dental cement. (23) Dental cement. (24). Black ink. (B) Surgical Procedure: 1) The skull is exposed by making an incision in the skin. (2) The PDMS window is positioned on the skull, and a reference outline is drawn for drilling. (3) The headbar is temporarily secured using glue. (4) Dental cement is applied over the headbar for stabilization. The black arrows denote the extended lines of the sagittal and coronal sutures, which serve as reference points for locating bregma. (5) The skull is drilled and thinned along the marked outline. (6) A metal plate is placed to ensure proper alignment for later PDMS compression. (7) The skull is removed to expose the brain. (8) The PDMS window is positioned over the exposed brain. (9) The PDMS window is pressed down until it aligns with the level of the skull. (10) The gap between the PDMS window and dental cement is sealed with thick glue. (11) The metal plate holding the PDMS window is removed. (12) A final layer of dental cement, mixed with black ink, is applied over the headbar, glue, and a small portion (∼0.1 mm) of the PDMS window. (C) PDMS window protection: (1) Uncovered view of the PDMS with metallic ring, and dorsal view of the whole mouse skull. The ring (1.8 cm diameter, 0.3 mm thickness) is secured with dental cement. The original position of the mouse skull is indicated in blue. (2) A protective ring and cap are used to safeguard the PDMS. (3) The PDMS is covered with the cap. (4) Uncovered view of the PDMS and brain without the ring and cap, and dorsal view of the whole mouse skull. (5) A layer of dry gauze is placed over the PDMS for protection. (6) A metal plate is secured over the gauze for additional protection.
12. Apply ophthalmic ointment (Figure 2A.4) to the eyes to prevent dryness and administer lidocaine to the shaved skin for local anesthesia (Figure 2A.2 and 5).
13. Administer carprofen (5–10 mg/kg) via subcutaneous injection for analgesia (Figure 2A.6).
14. Administer dexamethasone (8 mg/kg) via intramuscular injection into the hind limb to reduce brain inflammation prior to performing the craniotomy (Figure 2A.7 and 2C.7).
15. Expose the skull by incising the skin with surgical scissors (Figure 2A.8) and secure the skin laterally to the skull using Vetbond (Figures 2A.9 and 2B.1). A surgical scalpel (Figure 2A.10) may be used to assist in retracting the skin; however, avoid cutting the skin or muscle with the scalpel.
16. Place a PDMS window on the skull and use a black marker to draw a line indicating the area for drilling (Figure 2A.2). Ensure the PDMS window is smaller than the craniotomy.
17. Attach a headbar to the skull using super glue (Figures 2A.11, 12, and 2B.3).

***Note:*** The headbar should be positioned slightly towards the left side of the skull if securing the entire left hemisphere of the cortex is required. To achieve this, apply glue only to the upper right side of the headbar. The lower left part of the headbar should not contact the skull and should remain elevated. Ensure the headbar is parallel to the head’s orientation, as a tilted headbar may alter the animal's visual field during visual detection tasks later. In the example shown in Figure 2B.2, the approximate center of the visual cortex (A/P = 4.0 mm, M/L = 2.5 mm) is marked with a black dot.

10. Anesthetize the animal using isoflurane at a concentration of 3.0–4.0% for induction and maintain anesthesia at a concentration of 1.5%–2.0% throughout the surgical procedure.
11. Trim the fur at the surgical site with a hair clipper (Figure 2A.1) and disinfect the area using 70% ethanol followed by Povidone-iodine solution (Figure 2A.2 and 3).

18. Apply dental cement to the headbar (Figures 2A.13 and 2B.4).
19. At this step, roughly one hour into the surgery, administer a subcutaneous injection of saline (Figure 2B.14) to prevent hypovolemia. Continue administering saline every hour for the remainder of the surgery.
20. Drill along the previously drawn line until the skull is thin enough to move when touched (Figure 2B.5). Start with a 0.9 mm drill bit (Figure 2A.15) to outline the area, then switch to a 0.5 mm bit (Figure 2A.16) to refine and thin any remaining thick areas. Be sure to smooth the outer edge of the drilled region to avoid leaving any skull fragments attached post-removal.
21. Ensure that the metal plate intended to press the PDMS window later can be properly positioned and angled within the craniotomy (Figure 2B.6).
22. Apply cold saline.
  a. Carefully flip the drilled area of skull using tweezers (Figure 2A.17) to expose the brain (Figure 2B.7).
  b. Once the skull has been flipped, wipe the edge of the craniotomy with a triangular sponge (Figure 2A.18).

***Note:*** If there is bleeding, apply cold saline again.

**CRITICAL:** Have cold saline, cotton swab, and absorbable hemostatic gelatin sponges (Figure 2A.19 and 20) prepared in advance before flipping the skull in case of bleeding.

23. If bleeding occurs, wait for it to stop before proceeding. Then, place the PDMS window onto the brain (Figure 2B.8), ensuring it is clean. If not, wash the PDMS window with isopropyl alcohol and dry it before placement.
24. Press the PDMS window until it is either at the same level as the skull or slightly below it (Figure 2B.9).

**CRITICAL:** Do not apply excessive pressure to the PDMS window, as this can slow the animal's breathing rate, which can be fatal (troubleshooting 2). Continuously monitor the animal's respiration while pressing the PDMS window.

25. Fill the gap between the PDMS window and the skull with thick glue (Figure 2A.21), ensuring that the glue does not adhere to the metal plate used to press the PDMS window (Figure 2B.10, troubleshooting 3).
26. Slowly remove the metal plate that was used to hold the PDMS window in position (Figure 2B.11).
27. Apply dental cement over the glue, around the edge of the PDMS window, and on the headbar (Figure 2B.12).

***Note:*** In this example, black ink was mixed with the dental cement (Figure 2A.21, 22, and 23) for better protection from stray light for calcium imaging.

28. Place a metal ring (1.8 cm diameter, 0.3 mm thickness, 600 mg) on top of the headbar and secure it using dental cement (Figure 2C.1 and 2). Once the cement has fully hardened, place a black cover over the ring (Figure 2C.1 and 3). This procedure protects the PDMS window by minimizing its exposure during housing over several months (troubleshooting 4).

***Note:*** If a metal ring and black cover are unavailable, an alternative approach is to cover the PDMS window (Figure 2C.4) with dried gauze (Figure 2C.5) and secure it with a metal plate (Figure 2C.6). Ensure that the PDMS window is dry before placing the gauze.

### Virus or chemical injection protocol through the PDMS window

**Timing: ∼1–2 h**

**Figure 3:**
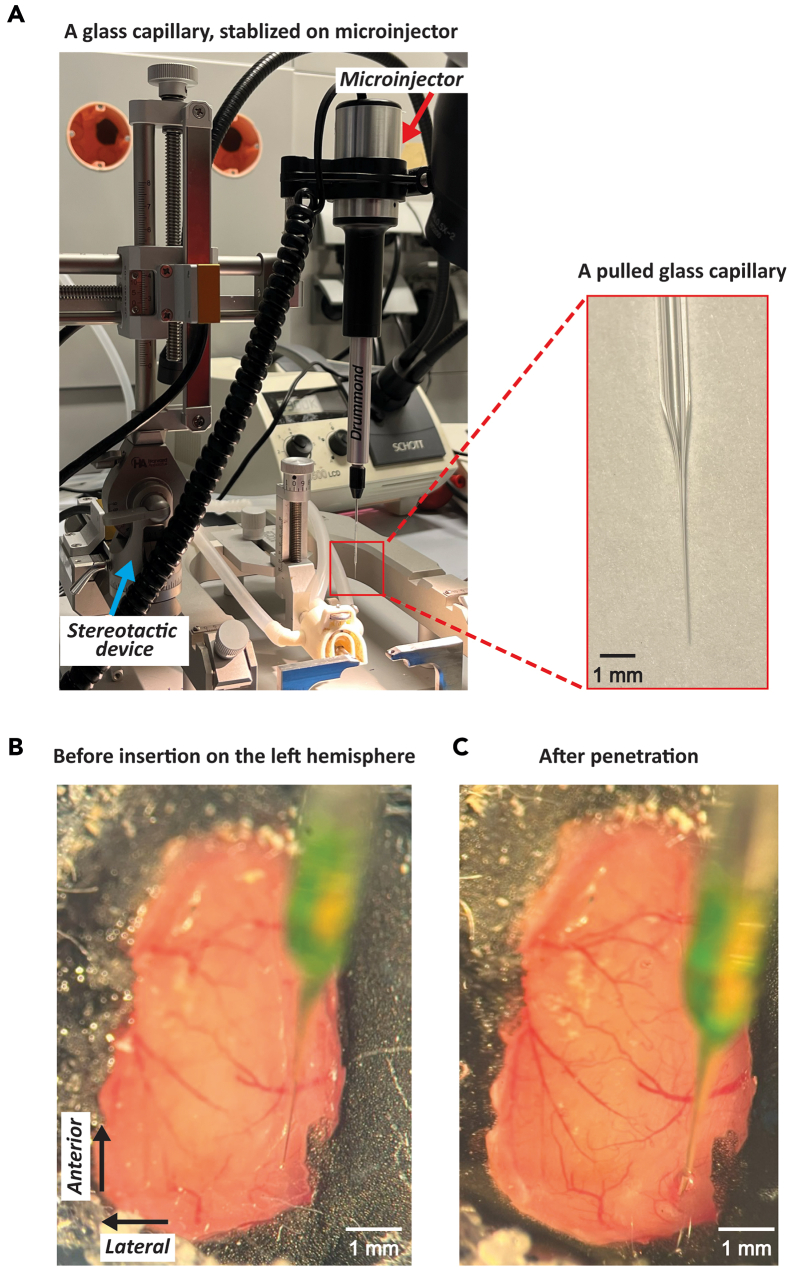
Virus injection procedure on PDMS (A) A glass capillary mounted on a microinjector within a stereotactic device. The enlarged image displays the tip of a glass capillary, with an approximate tip diameter of 100 μm and a base diameter of around 1 mm. (B) The capillary positioned above the PDMS prior to virus injection on the left hemisphere of the mouse brain. (C) The capillary penetrating the PDMS during the injection process.
31. Make a small incision on the surface of the PDMS window at the top of the region of interest (ROI) using a 30G needle, where the virus will be injected (Figure 3B).

***Note:*** This incision is necessary to facilitate the entry of the pulled glass capillary into the PDMS window, as the high surface tension of PDMS can impede insertion. In this example, the injection site in the visual cortex was localized based on results from wide-field calcium imaging. For experiments targeting a different brain region, we recommend indicating the location of bregma by marking the extensions of the sagittal and coronal sutures directly on the dental cement surface using a marker, as illustrated by the black arrows in Figure 2B.4. When the skull is later removed from one hemisphere (Figure 2B.11), these marks can assist in aligning anatomical features, such as blood vessels. When additional dental cement is applied subsequently (as shown in Figure 2B.12), the reference lines can be redrawn using a marker of a different color.

29. Use a puller to fabricate a thin tip (∼100 μm diameter) on a glass capillary for virus or chemical injection.
30. Fill the pulled glass capillary with the solution containing the necessary virus or chemicals, and attach it to the microinjector (Figure 3A). In this example, Adeno-Associated Viruses (AAV) were used, and Alexa Fluor 488 dye was included to visualize and verify the success of the injection.

**CRITICAL:** The incision does not need to extend all the way to the bottom of the PDMS window; a small superficial incision is sufficient.

32. Insert the pulled glass capillary into the small incision (Figure 3C).
33. Lower the capillary until it makes contact with the surface of the dura mater.

***Note:*** When the capillary touches the dura, the movement of the dura (or brain) becomes readily observable, allowing for the subsequent calculation of injection depth. This point is designated as 0 mm on the dorsal-ventral axis. If the brain is not clearly visible due to insufficient transparency of the PDMS window, the addition of demineralized water can enhance visualization.

**CRITICAL:** The use of tap water may lead to salt deposits on the PDMS window, possibly resulting in a foggy window. Once the PDMS window is contaminated, cleaning can be challenging. Thus, the use of tap water should be avoided.

34. Continue to lower the capillary until it punctures the dura mater. Upon puncturing the dura, a rebound effect can be observed as both the dura and brain return to their original positions due to the release of tension.
35. Position a glass capillary at the desired depth for injection.
36. Inject the viral suspension into the ROI and wait for approximately 10 min. If additional injections are necessary, repeat the procedure at various sites.
37. Remove the capillary slowly to minimize the risk of backflow of the injected virus.
38. Cover the PDMS window with a plastic cap or dried gauze and metal plate (Figure 2C.3 and 6).

## Expected outcomes

This protocol facilitates the detection of neural activity across various cortical regions of interest, leveraging the flexibility of PDMS fabrication in different sizes and shapes. A key advantage of this method is its ability to first monitor neural activity, such as through calcium imaging, in the context of specific experimental paradigms. Then, based on the imaging results, targeted virus or chemical injections can be performed in specific brain regions for precise manipulation, as the PDMS window allows easy penetration by a glass capillary, enabling precise viral or chemical injections. Notably, the PDMS window remains optically clear for several months (and up to a year) using this protocol. If deterioration occurs, a replacement procedure can be undertaken. To illustrate the capabilities of this method, we present the results of two experiments demonstrating reliable neural activity detection using wide-field and two-photon calcium imaging, as well as the feasibility of electrophysiological recordings combined with optogenetics through the PDMS window.

### Wide-field or two-photon calcium imaging recording through PDMS window

**Figure 4:**
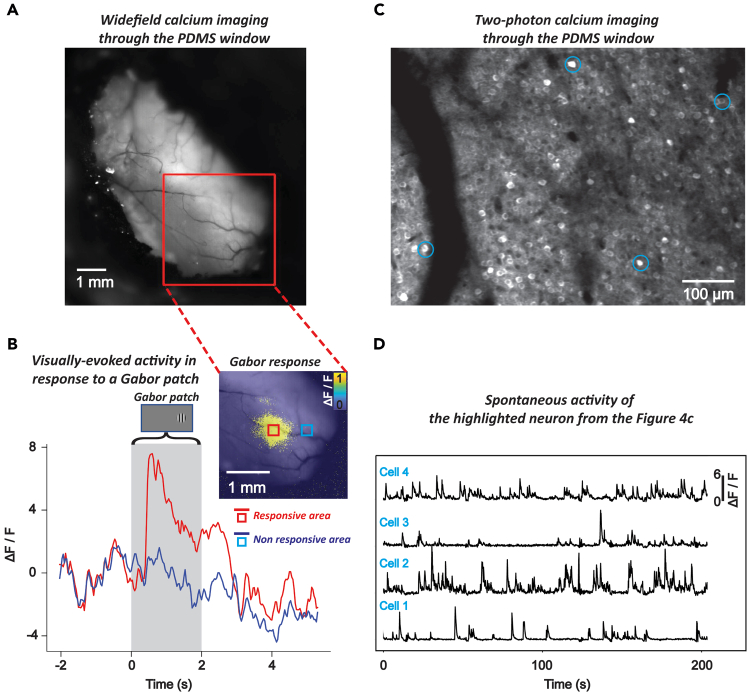
Wide-field and two-photon calcium imaging through a PDMS window (A) Wide-field calcium imaging through the PDMS window at the 43rd week after the PDMS implantation using a wide-field imaging camera, shown with the region of interest (red square) for calcium imaging in the left visual cortex. (B) Visually-evoked activity in response to the presentation of a Gabor patch. Left, top: An example of Gabor patch shown on a gray background in the right (contralateral) visual field. Right, top: Imaged neuronal response (ΔF/F) to the Gabor patch in the visual cortex, averaged over 40 trials. Each trial consisted of 2s pre-stimulation baseline, 2s visual stimulation, and 3s post-stimulation. Bottom: ΔF/F trace over time, averaged across 40 trials, from the neuronal region of interest indicated by the blue and red square in the top-right image. The red square marks the region that responded to the Gabor patch presentation, with its averaged neural response shown by the red line. The blue square marks the non-responsive region, with the blue line representing its corresponding neural activity. (C) Two-photon calcium imaging over the PDMS window. Example neurons selected for further analysis are marked with a blue circle. (D) Spontaneous activity of the highlighted neurons from the Figure 4C. The ΔF/F traces represent the normalized changes in fluorescence over a period of 200 s for the neuron.

By utilizing a transgenic animal expressing a calcium indicator, excitatory neuronal activity can be monitored through a PDMS window using both wide-field and two-photon calcium imaging. For instance, wide-field imaging was used to capture a clear view of the brain and its vasculature of a mouse over 10 months after implantation (Figure 4A). Neural activity was detected in the visual cortex of GCaMP6f-expressing mice (Rasgrf2-dCre x Ai148D) in response to visual stimuli, such as a moving bar (Methods video S1) or a Gabor patch (Figure 4B). While this example demonstrates the feasibility of detecting neural responses to visual stimuli, the experimental design can be adapted to study different sensory modalities, such as auditory stimuli, for which activation of the auditory cortex and distinct neuronal activity patterns would be expected. Since the PDMS window covers one entire cortical hemisphere, this enables simultaneous investigation of multiple cortical regions. Furthermore, neuronal activity can be also studied through the PDMS window using two-photon microscopy (Figure 4D and Methods video S2), for which we show recordings obtained one month (Figure 4C) and seven months (Figure 6B, right) after implantation. This methodology enables researchers to first identify regions of interest using wide-field imaging and subsequently perform high-resolution, single-neuron investigations within the same animal using two-photon microscopy, enabling a comprehensive analysis of cortical dynamics.

***Note:*** In the example illustrated in Figure 4A, a Gabor patch was presented on a gray screen with the following parameters: 40 trials, a diameter of 12 degrees, static gratings at a spatial frequency of 0.04 cycles per degree, 100% contrast. The images were captured using a wide-field imaging camera with a sampling frequency of 30 Hz and twofold pixel binning. Blue (470 nm, 5 mW, 15 Hz) and violet (405 nm, 5 mW, 15 Hz) LED light were alternately applied to the sample at each frame. In Methods video S1, a moving bar was used as the visual stimulus, with the following parameters: 25 trials, a stimulus speed of 10 cm/s, a flicker frequency of 6 Hz, a baseline duration of 3 s, and a target frame rate of 60 Hz. The images were also captured using a wide-field imaging camera with a sampling frequency of 30 Hz and twofold pixel binning, while blue (470 nm, 5 mW, 30 Hz) LED light was applied. For both examples, a screen with a width of 36 cm and a height of 21 cm was situated 11 cm away from the eye to the nearest point of the screen in the right visual field. In the example illustrated in Figures 4C and 4D, two-photon calcium imaging was conducted using a large field 2P microscope equipped with a 16× 0.8 NA objective (Nikon) and a digital zoom of 2×. Imaging was performed with an 80 MHz ultrafast pulsed laser at a fixed wavelength of 920 nm (Spark Lasers). Frames were acquired at a rate of 7.7 Hz using ThorImageLS software at a resolution of 1,024 × 1,024 pixels, covering a field of view of 973.54 μm × 973.54 μm. Preprocessing of two-photon calcium imaging was performed using the automated segmentation algorithm in Suite2p for neuron detection.

Methods video S1. Example of neuronal responses to moving bar visual stimuli recorded via widefield calcium imaging, related to the section of wide-field or two-photon calcium imaging recording through PDMS window in expected outcomes The left panel depicts averaged calcium activity using a colormap scale, whereas the right panel shows the averaged calcium signals in the original video format. The upper right image depicts the anatomical reference, with the red square indicating the region of interest selected for calcium imaging analysis.

Methods video S2. Example of neuronal activity recorded at resting state using two-photon microscopy, related to the section of wide-field or two-photon calcium imaging recording through PDMS window in expected outcomes White sparks represent neural activity events.

### Optogenetics and electrophysiology recording through PDMS window

**Figure 5:**
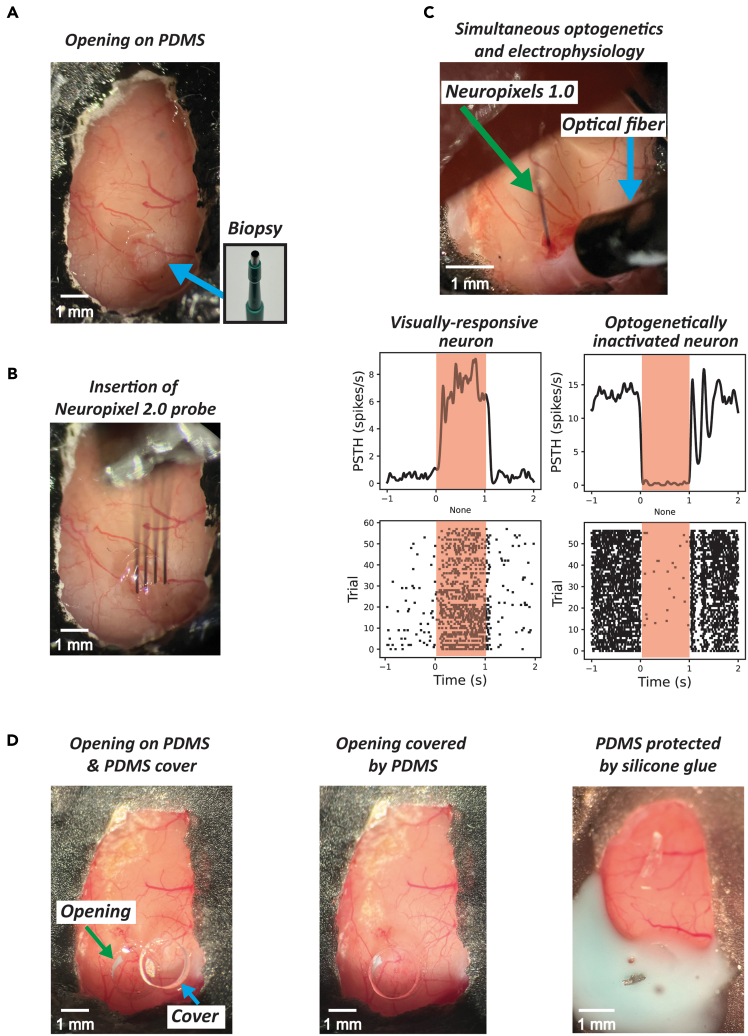
Optogenetic manipulation and electrophysiological recording via the PDMS window (A) Example of an opening performed on a PDMS window using a biopsy punch. (B) Insertion of a Neuropixels 2.0 probe through the window opening. (C) Simultaneous optogenetics and electrophysiology over the PDMS window. A Neuropixels 1.0 probe was used for electrophysiological recording over the PDMS window. The bottom left panel illustrates neural responses to visual stimuli, while the bottom right panel depicts neuronal inhibition. Red shaded areas indicate the period of sensory or optogenetic stimulation, respectively. (D) Sealing procedure following electrophysiological recordings. The left panel displays an exposed opening in the PDMS with a corresponding PDMS cover positioned adjacent to it. The middle panel displays the opening after being sealed with the PDMS cover. The right panel displays the application of two-component silicone glue (Twinsil) over the sealed area to ensure additional protection.

Following calcium imaging to identify the region of interest (ROI) for investigation through the PDMS window, two additional methodologies can be further employed. The first approach involves electrophysiological recordings to examine neuronal spiking activity in the identified ROI. Electrophysiology serves as an alternative and as a complementary method to two-photon imaging for assessing neuronal spiking activity, offering substantially higher temporal resolution. A key advantage of PDMS over glass windows is their versatility: researchers can either perform an opening in the PDMS or directly penetrate it with an electrode. However, certain delicate electrodes, such as Neuropixels,^19^^,^^20^ cannot be directly inserted through PDMS. In such cases, an opening is necessary (Figure 5A). In our study, we successfully inserted Neuropixels 1.0^19^ and 2.0^20^ probes by creating a 1.5 mm opening in the PDMS window using a biopsy punch (Figures 5B and 5D left). The second approach utilizes the optical transparency of PDMS for optogenetic manipulation. Once calcium imaging has identified the ROI based on the experimental design, viral vectors can be injected into the target area following the protocol detailed in Figure 3. After viral expression, optogenetic stimulation can be effectively applied, as PDMS is transparent, allowing light to penetrate effectively. Moreover, electrophysiology and optogenetics can be combined to causally manipulate and at the same time monitor neuronal responses (Figure 5C, top). As an example, we demonstrate neuronal activation in the visual cortex in response to visual whole-screen gratings (Figure 5C, bottom left) and concurrent neuronal inhibition through optogenetic manipulation (Red light laser with 637 nm wavelength and 8 mW intensity) in a mouse expressing Jaws (mediated by injection of AAV8-Syn-Jaws) (Figure 5C, bottom right). Once the electrophysiology recording is completed, the opening should be sealed by placing a circular piece of PDMS over it (Figure 5D middle). This ensures the maintenance of brain health by preserving moisture and preventing desiccation. The circular piece of PDMS extracted during the biopsy procedure should be reused here, to ensure a tight fit. Otherwise, a new piece can be easily prepared by extracting it from a PDMS plate using a biopsy punch. While a PDMS cover alone may not stay in place, applying a two-component silicone glue (e.g., Twinsil) around the perimeter of the opening can help secure it (Figure 5D right). When sealing the PDMS window with silicone, it is crucial to leave some portion of the window uncovered, as shown in Figure 5D right. Fully covering the PDMS can result in strong adhesive bonding, making removal difficult and risking detachment of the entire window. To reduce this risk, a minimal amount of silicone should be used, ensuring that some part of the PDMS remains uncovered. Based on our experience, covering less than half of the PDMS window with silicone is optimal for ease of removal. However, even when three-quarters is covered, the window can typically still be removed successfully if handled carefully.

***Note:*** In the example illustrated in Figure 5C, a full-screen grating was presented for 1 s with the following parameters: a spatial frequency of 0.04 cycles per degree, a temporal frequency of 2 Hz, and an inter-trial interval of 3 to 8 s. Consistent with the calcium imaging setup, a screen with a width of 36 cm and a height of 21 cm was also situated 11 cm away from the eye to the nearest point of the screen in the right visual field. Neuropixels data were acquired using the Open Ephys software platform. The recorded data were subsequently processed using Kilosort3 for spike sorting.

### Performance of PDMS windows over time

**Figure 6:**
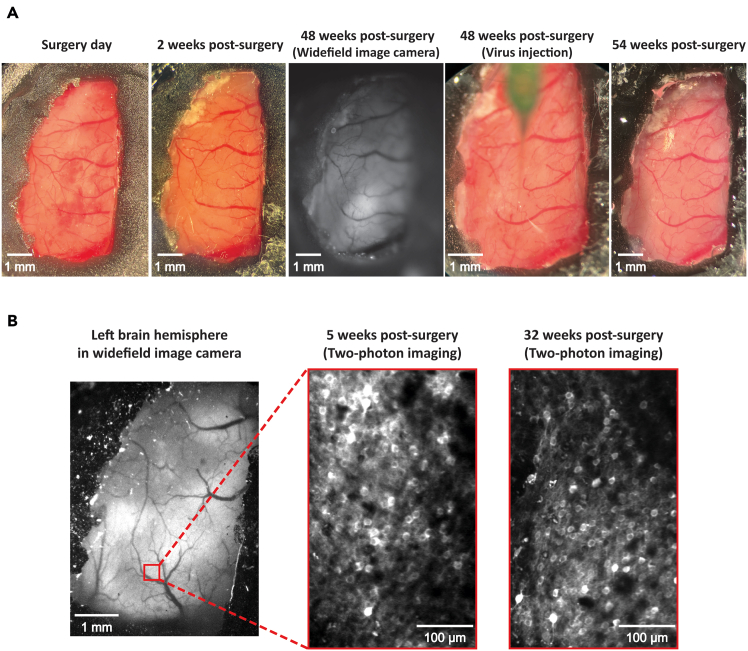
Tracking PDMS window quality over time (A) Longitudinal monitoring of the PDMS window in the same animal from the day of surgery up to one year. The images, from left to right, depict: the day of surgery, 2 weeks post-surgery, 48 weeks post-surgery on the day of calcium imaging using a wide-field camera, 48 weeks post-surgery during virus injection, and 54 weeks post-surgery. (B) Longitudinal assessment of two-photon calcium imaging. Left: Image of the entire left hemisphere of the cortex by wide-field imaging camera, with the red square highlighting the region of interest for two-photon imaging. Middle: Two-photon calcium imaging at 5 weeks post-surgery. Right: Two-photon calcium imaging at 32 weeks post-surgery. Note: We did not follow the same group of neurons across multiple months here since the primary goal of our experiments was not long-term tracking.

While a prior study mentioned that skull regrowth could take place between 5 and 8 months following the implantation of a PDMS window,^11^ our protocol enables PDMS windows to retain their optical transparency for extended periods, ranging from several months to a year (Figure 6A). This enabled us to successfully perform both wide-field and two-photon calcium imaging over the extended timeframe as indicated by earlier results (Figures 4A and 6B). This extended functionality may be due to the flexible design of our custom-made window and refined surgical techniques (Figures 1 and 2). In our protocol, the PDMS window can be easily shaped into any form or size by using scissors, a biopsy punch, or other comparable instruments (Figure 1). PDMS is flexible and bendable, which is advantageous and well-suited for covering large brain areas with uneven surfaces. When the cranial window is large, rigid materials such as glass may leave gaps between the window and the brain surface due to the brain’s curvature. This curvature can result in insufficient and uneven pressure on the dura during window placement, which can promote dura or skull regrowth over time.^21^ In contrast, flexible materials such as PDMS adapt to the brain’s contours, ensuring a snug and secure fit. However, achieving this requires precise surgical techniques rather than simply placing the material on the brain. Otherwise, skull regrowth may occur earlier. Although degradation in the window’s quality is occasionally observed (∼2%–3% of total animals), replacement of the window is feasible with our protocol (see troubleshooting section and Figure 8). Through our experience, we have identified the cleaning procedure as a critical factor influencing the long-term optical quality of PDMS used in calcium imaging experiments. When the PDMS surface appears unclean, it is necessary to clean it prior to imaging to ensure high-quality image acquisition. However, using water or saline for this purpose should be avoided. Although a single instance of cleaning with these solutions may not noticeably affect the material, repeated use of water or saline solutions leads to a gradual decline in transparency of PDMS over time. To maintain the optical clarity of PDMS, we recommend using isopropyl alcohol for cleaning. Importantly, the method of application should be carefully controlled. Pouring isopropyl alcohol directly onto the PDMS surface can allow the solvent to infiltrate small gaps between the PDMS and dental cement, posing a potential toxic risk to the underlying brain tissue. To reduce this risk, we suggest lightly moistening a sponge or tissue with isopropyl alcohol and gently wiping the PDMS surface to ensure safe and effective cleaning. In addition to degradation of window quality, replacement may occasionally be required due to unforeseen issues, such as the emergence of air bubbles between the brain and the window. Although such instances are rare, our experience indicates that approximately 5% of animals may require window replacement surgery.

### Comparison with other window types

**Figure 7:**
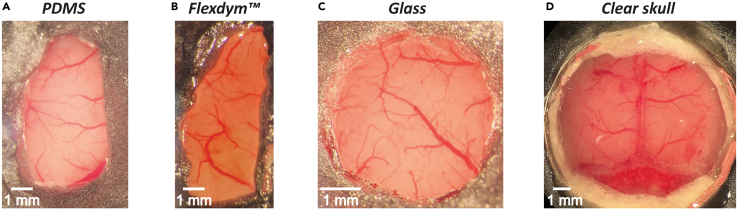
Examples of different window materials: PDMS, Flexdym, glass, and clear skull

In calcium imaging studies, researchers typically employ either clear skull procedures or cranial windows typically made of glass or rigid polymers, depending on the imaging resolution required. Clear skull procedures are commonly used for wide-field calcium imaging,^1^^,^^9^^,^^22^ as this technique does not demand high-resolution visualization. In contrast, the visualization of individual neurons requires a highly transparent window, making glass windows the preferred option for chronic two-photon imaging.^8^^,^^15^^,^^23^^,^^24^ Although glass windows provide excellent optical clarity, their rigidity and flat geometry limit their suitability for covering larger cortical areas. This limitation is generally acceptable when targeting small, relatively flat regions of the brain, but becomes problematic when attempting to cover larger areas due to the brain’s natural curvature. To overcome this limitation, recent studies have proposed the use of polymeric materials as alternative cranial window substrates,^12^^,^^13^ enabling more effective coverage across wider brain regions. While these studies have demonstrated that polymeric materials can provide excellent optical clarity for neuronal imaging, many of these materials are not well-suited for procedures requiring glass capillary insertion or the creation of openings for electrophysiology. Materials such as polyethylene terephthalate^12^ or polymethylpentene films,^13^ used in the earlier studies, are often too rigid to allow such interventions once implanted on the brain. To overcome this limitation, we employed PDMS, softer polymeric material, that allow easier penetration to accommodate these experimental needs. To provide an assessment of the results that can be expected through the use of PDMS windows, we compared four types of approaches and materials: clear skull, PDMS, Flexdym, and glass (Figure 7; Table 1). Here, we do not expect a significant difference in optical clarity between PDMS windows and other rigid materials, such as glass or Flexdym. Flexdym (250 μm thickness, 15 kN/m tear strength, Eden Tech) is a commercially available material proposed as an alternative to PDMS. This qualitative comparison is based on the experience gained by the authors during the past decade when performing experiment with the different types of windows discussed here. Each window type presented distinct advantages and limitations. In terms of optical clarity, PDMS, Flexdym and glass windows provide the best visibility, allowing clear visualization of both large and small blood vessels. While the clear skull procedure also enables observation of large and some small vessels, its clarity is inferior compared to PDMS, Flexdym and glass windows. Furthermore, the transparency of clear skulls declines over time, often becoming foggy within several months. In contrast, PDMS, Flexdym and glass windows maintain their transparency for longer durations, ranging from several months to a year (Figure 6). The surgical implantation of PDMS, Flexdym and glass windows, however, is more technically challenging and carries greater risk compared to clear skull procedures, as it requires removal of the skull. In contrast, clear skull procedures are entirely non-invasive, relying solely on the use of various glues.

**Table 1:** Qualitative comparison of the optical properties of different window materials: PDMS, Flexdym, glass, and clear skull

Category	PDMS	Flexdym	Glass	Clear skull
Clearness	Both large and small blood vessels are clearly visible (Figure 7A)	Both large and small blood vessels are clearly visible (Figure 7B)	Both large and small blood vessels are clearly visible (Figure 7C)	Both large and smaller vessels are visible after surgery but not as transparent as other materials (Figure 7D)
Stability Over Time	Relatively stable over a prolonged period	Relatively stable over a prolonged period	Relatively stable over a prolonged period	Quality degrades after several months
Surgical Difficulty	Difficult	Difficult	Difficult	Easy
Replacement After Degradation	Suitable	Suitable	Suitable	Not feasible
Flexibility of Window Shape	Any shape	Any shape, though more challenging to cut	Restricted to predefined shapes (e.g., circular)	n.a.
Compatibility with Viral Injection	Compatible	Not compatible	Not compatible	Requires craniotomy
Electrophysiology	Requires craniotomy	Not compatible	Not compatible	Requires craniotomy
Wide-field Calcium Imaging / Optogenetics	Suitable	Suitable	Suitable	Suitable
Two-Photon Imaging	Suitable	Suitable	Suitable	Not suitable

Among PDMS, Flexdym and glass windows, PDMS and Flexdym provide greater versatility in terms of size and shape customization. Glass windows require advance preparation, with predetermined shapes such as circular or rectangular designs. Conversely, PDMS and Flexdym windows can be customized on the day of surgery by cutting the material with scissors, a biopsy punch, or similar tools (Figure 1D). This adaptability makes PDMS and Flexdym particularly advantageous for studies requiring coverage of large brain areas, such as those focused on multisensory integration between the auditory and visual cortices^25^ or sensory-motor transformations.^6^^,^^7^ In particular, PDMS is particularly well-suited and more advantageous for multimodal approaches that require additional invasive procedures such as viral injections for optogenetics or electrode insertions for electrophysiology. In such cases, using a glass window would require drilling, which is impractical as it disrupts high-quality optical access. Recent studies have explored the use of polymer-based materials.^12^^,^^13^ However, not all polymeric materials are compatible for applications that require mechanical access to the brain such as the insertion of a glass capillary or the creation of an opening. For instance, Flexdym is difficult to penetrate once implanted, since the material is more rigid than PDMS. In contrast, PDMS allows easy insertion of tools such as metal electrodes^11^ or pulled glass capillaries for viral injections (Figure 3C). A notable limitation of PDMS (and of all other window types as well) is its incompatibility with insertion of Neuropixels probe, which are increasingly popular for recording neuronal activity across numerous channels. Neuropixels probes are unable to penetrate PDMS, as they tend to bend and risk breaking. A previously published method involves creating a 3D-printed mold with predefined holes for probe insertion.^1^ However, this approach lacks both sufficient optical clarity for calcium imaging as well as flexibility in probe insertion sites, as the holes must be created at predefined insertion locations before window implantation. Thus, this approach restricts the flexibility of insertion sites when the goal is to first identify the insertion areas through calcium imaging and then insert the probe. However, small openings can be made in PDMS using a biopsy punch, enabling Neuropixels probe insertion after identifying the region of interest through calcium imaging (Figures 5A and 5B).

## Limitations

Neuropixels probes (and other silicon probes) cannot be directly inserted into polydimethylsiloxane (PDMS), whereas more rigid materials such as metal electrodes and glass pipettes can be inserted without issue. This presents a challenge, especially given the remarkable progress made in neurotechnology over the past few years. However, small openings can be made in PDMS windows to enable this type of recordings (see optogenetics and electrophysiology recording through PDMS window section and Figure 5A).

## Troubleshooting

### Problem 1

PDMS not being fully transparent (related to steps 1–9).

### Potential solution

If inappropriate materials, such as 3D-printed molds, are used for PDMS synthesis, issues may arise. Specifically, two primary problems can occur when PDMS is poured into such molds or other non-smooth materials: (1) incomplete curing, resulting in a partially liquid state, and (2) a lack of full transparency due to the mold’s uneven or rough surface. In our initial attempts, PDMS failed to solidify properly when an unsuitable mold was used. To ensure successful curing and transparency, it is essential to select an appropriate mold material. A Petri dish, as recommended in the materials section, provides a smooth, flat surface that ensures PDMS cures properly and remains transparent. Thus, we do not recommend using containers other than Petri dishes, as the surface of alternative containers may not be sufficiently smooth for PDMS synthesis.

### Problem 2

The animal’s breathing rate slows down when applying pressure to the PDMS with a metal plate (related to Step 24).

### Potential solution

If the animal experiences difficulty breathing during the surgical procedure when pushing the PDMS window, which could potentially be life-threatening, it indicates that too much force is being applied. In such cases, it is crucial to reduce the pressure and proceed with more care to avoid causing harm to the animal.

### Problem 3

PDMS becomes dirty after being placed over the brain on the day of the surgery (related to Step 25).

### Potential solution

**Figure 8:**
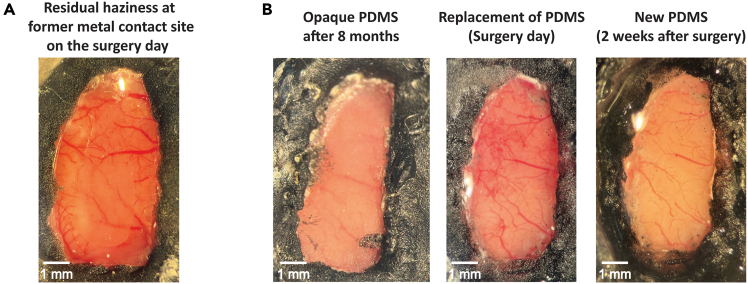
Potential complications following PDMS window placement (A) An example of residual haziness in the posterior region of the PDMS window at the location of the former metal contact, observed on the surgery day (see Figure 2B.9 and 10). (B) Replacement of an opaque PDMS window after 8 months. The left image shows an opaque PDMS window, the middle image shows the window immediately after replacement on the day of surgery, and the right image shows the clear brain following natural dissipation of residual blood after two weeks.

As shown in Figure 8, PDMS can sometimes appear not fully transparent, especially around the area where the metal plate was applied (Figures 2B.9, 2B.10, and 7A). Three potential reasons why PDMS may become dirty are: 1) the metal plate was dirty, 2) PDMS was over-washed or left in ethanol or isopropyl alcohol for an extended period, or 3) glue from the metal plate transferred onto the PDMS during the surgical procedure. To prevent these issues, it is recommended to thoroughly clean the metal plate with isopropyl alcohol before starting the PDMS placement surgery. The PDMS window should also be rinsed with isopropyl alcohol and fully dried before being placed on the brain. However, prolonged exposure to isopropyl alcohol should be avoided as it may negatively affect PDMS transparency. Lastly, avoid allowing glue to come into contact with the metal plate, as it is difficult to clean once it has adhered.

### Problem 4

PDMS loses its transparency after an extended period, usually several months (related to Step 28).

### Potential solution

PDMS typically maintains its clarity for about a year, although it may sometimes lose its transparency over time. If the window becomes excessively opaque (Figure 7B, left), it can be replaced relatively easily through an additional surgical intervention. The procedure begins by drilling away the edge of the dental cement around the PDMS window. Once this is done, the PDMS can be removed using tweezers, making sure to remove any remaining dental cement waste. Incomplete removal of cement may cause the PDMS to tear during extraction, making the process more difficult. After the PDMS has been removed, the cranial window should be thoroughly cleaned to remove any remaining cement debris. Subsequently, one can follow the surgical steps detailed in Figure 2B.7-12 to insert a new window. Finally, the area is allowed to heal for 1–2 weeks, during which time any residual blood will naturally dissipate as shown in Figure 7B, right.

### Problem 5

An opening cannot be made in the PDMS window with precision, resulting in an uneven or poorly defined shape (Figure 5).

### Potential solution

Creating an opening in the PDMS window can be difficult when using just a scalpel or needle, because of the material’s soft and pliable nature. This makes it challenging to achieve a precise incision. As of now, the most reliable method for performing this procedure is by using a biopsy needle, which offers greater control and precision when cutting through the PDMS window. It is recommended to avoid using extremely sharp instruments such as a scalpel or needle.

## Resource availability

### Lead contact

Further information and requests for resources and reagents should be directed to and will be fulfilled by the lead contact, Umberto Olcese (u.olcese@uva.nl).

### Technical contact

Technical questions on executing this protocol should be directed to and will be answered by the technical contact, Kengo Takahashi (k.takahashi@uva.nl).

### Materials availability

All information on the materials utilized in this study is provided within this article. For further information, please reach out to the lead (u.olcese@uva.nl) or technical (k.takahashi@uva.nl) contact.

### Data and code availability

Original data have been deposited to Zenodo: https://doi.org/10.5281/zenodo.15577657.

## Acknowledgments

This project was made possible through the support of a grant from the 10.13039/501100011730Templeton World Charity Foundation, Inc. (TWCF0646) to R.Y., U.O., and C.M.A.P. and a grant from the European Union (EIC Pathfinder project NAP, grant agreement 101099310) to U.O. The opinions expressed in this publication are those of the authors and do not necessarily reflect the views of Templeton World Charity Foundation, Inc. (https://www.templetonworldcharity.org/). The funders had no role in study design, data collection and analysis, decision to publish, or preparation of the manuscript. S.P.Q. and R.Y. also acknowledge support from the 10.13039/100000065NINDS (RM1NS132981) and 10.13039/100000053NEI (R01EY035248). We would also like to acknowledge administrative support by Reinder Dorman, technical assistance for PDMS production by Dr. Paola Fanzio, implementation of GCaMP expression methods by Mathis Bassler and Lilian Emming, and the technical contribution of the Technology Center of the University of Amsterdam.

## Author contributions

Conceptualization, K.T. and U.O.; methodology, K.T. and G.J.H.; investigation, K.T., G.J.H., D.B., J.-Y.W., and S.P.Q.; writing – original draft, K.T.; writing – review and editing, K.T., G.J.H., D.B., J.-Y.W., S.P.Q., R.Y., C.M.A.P., and U.O.; funding acquisition, R.Y., C.M.A.P., and U.O.; supervision, U.O.

## Declaration of interests

The authors declare no competing interests.
